# A Scoping Review of Tailored Self-management Interventions among Adults with Mobility Impairing Neurological and Musculoskeletal Conditions

**DOI:** 10.3389/fpubh.2016.00165

**Published:** 2016-09-12

**Authors:** Matthew Plow, Sabrina Mangal, Kathryn Geither, Meghan Golding

**Affiliations:** ^1^Frances Payne Bolton School of Nursing, Case Western Reserve University, Cleveland, OH, USA

**Keywords:** disability, self-care, personalization, health behavior, chronic disease, tertiary prevention

## Abstract

A critical public health objective is to optimize and disseminate self-management interventions for the 56.7 million adults living with chronic disabling conditions in the United States. A possible strategy to optimize the effectiveness of self-management interventions is to understand how best to tailor self-management interventions to the needs and circumstances of each participant. Thus, the purpose of this scoping review was to describe randomized controlled trials (RCTs) of tailored self-management interventions in adults with neurological and musculoskeletal conditions that characteristically result in mobility impairments. The 13 RCTs included in the scoping review typically compared tailored interventions to non-tailored interventions or usual care among adults with chronic pain, stroke, and/or arthritis. The tailored interventions were diverse in their delivery formats, dosing, behavior change techniques, and tailoring strategies. We identified 13 personal characteristics (e.g., preferences and theoretical constructs) and 4 types of assessment formats (i.e., oral history, self-report questionnaires, provider-reported assessments, and medical records) that were used to tailor the self-management interventions. It was common to tailor intervention content using self-report questionnaires that assessed personal characteristics pertaining to impairments and preferences. Content was matched to personal characteristics using clinical judgment or computer algorithms. However, few studies adequately described the decision rules for matching content. To advance the science of tailoring self-management interventions, we recommend conducting comparative effectiveness research and further developing a taxonomy to standardize descriptions of tailoring. We discuss the opportunities that are now coalescing to optimize tailored self-management. We also provide examples of how to merge concepts from the self-management literature with conceptual frameworks of tailoring from the health communication literature.

## Introduction

A critical public health objective is to optimize and disseminate self-management interventions for the 56.7 million adults living with chronic disabling conditions in the United States (1, 2). Adults with chronic musculoskeletal and neurological conditions often experience a variety of impairments and mobility problems that interact with environmental factors to create barriers for self-managing symptoms and engaging in healthy behaviors (3–8). Difficulties with engaging in physical activity, developing healthy eating habits, and taking medications as prescribed can independently and cumulatively increase the risk of secondary conditions (e.g., cardiovascular disease), accelerate functional declines, and reduce quality of life (7–9). The goal of a self-management intervention is to support the learning of skills (e.g., problem solving and resource utilization) that facilitate engagement in healthy behaviors and improve quality of life (10). However, the effectiveness of self-management interventions to promote sustained behavior change is variable and may not be equally effective among all adults with musculoskeletal and neurological conditions (11–14).

A possible strategy to improve the effectiveness of self-management interventions across all adults with musculoskeletal and neurological conditions is to understand how best to tailor self-management interventions to the needs and circumstances of each participant. Tailoring means altering information, delivery strategies (e.g., at a distance using computer tailoring or in person using clinical tailoring), and/or dosing (i.e., the frequency or amount of contacts) based on an assessment of an individual’s specific characteristics (e.g., psychology, biomarkers, and/or environment) related to the outcome of interest (15, 16). Patients prefer tailored interventions and view them as being more relevant to their needs (17). Thus, patients may be more likely to think about and adhere to tailored interventions (16, 18, 19). Nonetheless, research indicates that tailored interventions are only slightly more effective than non-tailored interventions in promoting healthy behaviors (19–21).

Scholars agree that identifying optimal methods and establishing a framework to standardize terminology may improve the effectiveness of tailoring (15, 16, 22). Hawking et al. (15) recently proposed such a framework. Rather than viewing an intervention as tailored or non-tailored, Hawking et al. suggest that tailoring is on a continuum of degrees. The degree to which participants are divided into homogenous groups is called *segmentation*, while the degree to which the information is consistent with individual characteristics is called *customization*. Information can be segmented and customized in three main ways: (1) conveying how the intervention is designed specifically for the participant (i.e., personalization), (2) presenting information about one’s self (i.e., feedback), and (3) using biological and/or psychological characteristics (i.e., content matching). A tailored self-management intervention, for example, could personalize content by explaining how information is relevant to a person’s chronic condition (i.e., personalization), presenting information on health status changes (i.e., feedback), and providing persuasive messages based on a measured theoretical construct, such as self-efficacy (i.e., content matching).

Although the research literature on tailoring self-management interventions is growing, several factors have impeded its growth. First, the term *tailoring* is used inconsistently within the self-management literature. For example, researchers use the term tailoring to describe adapting an intervention for a specific clinical population. Second, self-management interventions may not be labeled as self-management interventions even though the goal of the intervention is to promote healthy behaviors among adults with disabling conditions (23). Third, self-management research is often conducted within disciplinary silos that are either specific to a chronic condition or to a particular health care profession, which is conducive to expanding research, but not necessarily to advancing research.

To avoid reinventing the wheel in developing and testing tailored self-management interventions, it is important to review the existing research. To date, no review articles have focused on tailored self-management interventions among adults with neurological and musculoskeletal conditions. Thus, the purpose of this scoping review was to describe randomized controlled trials (RCTs) of tailored self-management interventions in adults with neurological and musculoskeletal conditions that characteristically result in mobility impairments. Specifically, we focused on summarizing the outcomes of these RCTs and the strategies used to promote behavior change. We used a published taxonomy (24) and standardized definitions (15, 22) to summarize the behavior change techniques and strategies used to tailor self-management interventions.

## Methods

A scoping review is a relatively new research methodology, but its use and recognized utility is growing. It involves summarizing, synthesizing, and analyzing the existing literature and is intended to provide clarity about a specific topic and to identify needed research (25). The purpose of a scoping review is not to evaluate the quality of evidence and come to a conclusion about the level of evidence for a causal relationship, as in a systematic literature review. Instead, the goal of a scoping review is to summarize the literature by time, location, and origin (25, 26). Arksey and O’Malley (27) outline five steps in a scoping review: (1) identifying the research question and operationalizing the definitions; (2) identifying relevant studies through electronic databases and reference lists; (3) establishing inclusion–exclusion criteria for the selection of studies; (4) charting the data through a narrative review; and (5) collating, summarizing, and reporting the results.

### Step 1: Identify Research Question and Operationalize Definitions

The questions we aimed to answer from the scientific literature were: (1) What are the conclusions that researchers have drawn from conducting RCTs of tailored self-management interventions in adults with neurological and musculoskeletal conditions? (2) How are tailored self-management interventions being implemented (e.g., delivery format and dosing) and examined (e.g., research design and outcome measures) in adults with neurological and musculoskeletal conditions? (3) What are the specific behavior change techniques and tailoring strategies being implemented in these self-management interventions?

To answer the last question, we used the Coventry Aberdeen London – Refined (CALO-RE) taxonomy (24) and Hawkins et al.’s definitions to describe tailoring strategies (15). CALO-RE defines 40 behavior change techniques (e.g., goal setting, using a role model, and enlisting social support) commonly used in intervention research. CALO-RE is based on the Abraham and Michie’s taxonomy (28), which was found to have good consistency between coders and between intervention manuals and research articles. CALO-RE improves upon the original taxonomy by further clarifying and delineating behavior change techniques.

We used Hawkin et al.’s (15) definition of personalization (i.e., identification, raising expectation of customization, and contextualization), feedback (i.e., descriptive, comparative-normative, comparative-progress, and evaluative), and content matching (i.e., the variables used to tailor content) to describe how the self-management interventions were tailored. We elaborated on Hawkin’s definition of content matching using a qualitative approach. Specifically, categories were developed to describe the assessment format and the type of participant characteristics that were used to tailor the self-management intervention (i.e., providing details on content matching).

To develop the categories, we first extracted relevant paragraphs from each included article that described how the self-management intervention was tailored. We then reviewed each description to identify similarities to develop categories with definitions. Finally, we coded and counted the frequency each time that an intervention strategy was consistent with a particular category.

### Step 2: Identify Relevant Studies

We used multiple search strategies to identify studies on tailored self-management interventions in adults with neurological and musculoskeletal conditions. The databases used to complete the search included PubMed, the Psychology and Behavioral Sciences Collection, CINAHL, and Google Scholar. We used the following MeSH and/or subject terms: disabled persons, nervous system diseases, musculoskeletal diseases, sensory disorders, spina bifida, traumatic brain injury, polio, stroke, lupus, muscular dystrophy, Parkinson, mobility, autoimmune disease, neuromuscular, arthritis, cerebral palsy, amputation, fibromyalgia, spinal cord injury, and multiple sclerosis. We combined these terms with: tailor*, behavior*, personalization, individualized, rehabilitation, self-care, self-management, patient-centered, and education. The search was limited to English and human adults ≥18 years of age. In addition, we searched the reference list of relevant review articles.

### Step 3: Eligibility Criteria

Study criteria were implemented to ensure that we reviewed only RCTs of tailored self-management interventions in adults with neurological and musculoskeletal conditions. We defined self-management interventions broadly to include any type of health education that (a) imparted health-related information that influences values, beliefs, attitudes, and motivations; (b) achieved health- or illness-related learning through knowledge acquisition, assimilation, and dissemination; and (c) led to the development of skills or lifestyle/behavior modification (29). To be considered as tailored, there needed to be a description of an assessment used to alter and match the content of the intervention to at least two different characteristics of the participant. Table 1 summarizes the inclusion–exclusion criteria.

**Table 1:** **Study criteria**.

Inclusion criteria	(1) Randomized controlled trial of a tailored self-management intervention (2) Community-dwelling adults who acquire diseases or impairments in neurological or musculoskeletal systems that characteristically results in physical disability, problems with mobility, and/or chronic pain and fatigue (3) Included an outcome measure of medication adherence, physical activity, nutrition, sleep hygiene, smoking cessation, or alcohol use (4) Described in the English language (5) Published between 1980 and 2015
Exclusion criteria	(1) Studies primarily evaluating the beneficial effects of exercise programs, medications, or vocational rehabilitation programs (2) Studies including children or adolescents under 18 years old, adults living in a nursing home or receiving the entire intervention during inpatient care, older adults without needing to have a condition as defined above, and adults with a primary diagnosis of cardiovascular disease, epilepsy, cancer, endocrine disease, mental health disorder, or Alzheimer’s disease (3) Studies on motivational interviewing (4) Conference proceedings, abstracts, and review articles

#### Search Procedure

The search procedure was divided into two phases: (1) title and abstract review and (2) full-text article review. For the first phase, we scanned titles and abstracts to identify any potential study that described intervention strategies to promote healthy behaviors. We also excluded reviews and research protocols, studies published before 1980, non-randomized studies, and studies of children and healthy adults. For the second phase of review, we scanned the articles in detail to code behavior change techniques and excluded studies that did not meet any remaining criteria.

### Step 4: Charting Data

Sample characteristics (e.g., gender, race/ethnicity, and function), the type of research design, outcome measures, and intervention characteristics (i.e., delivery format, behavior change techniques, tailoring strategies, and the three dosing parameters of duration, frequency, and amount) were extracted from the articles. The second and third author extracted data and coded behavior change techniques independently. Behavior change techniques were compared and tallied across studies. Disagreements in coding were discussed with the first and last author until consensus was reached.

## Results

### Step 5: Collating, Summarizing, and Reporting Results

**Figure 1:**
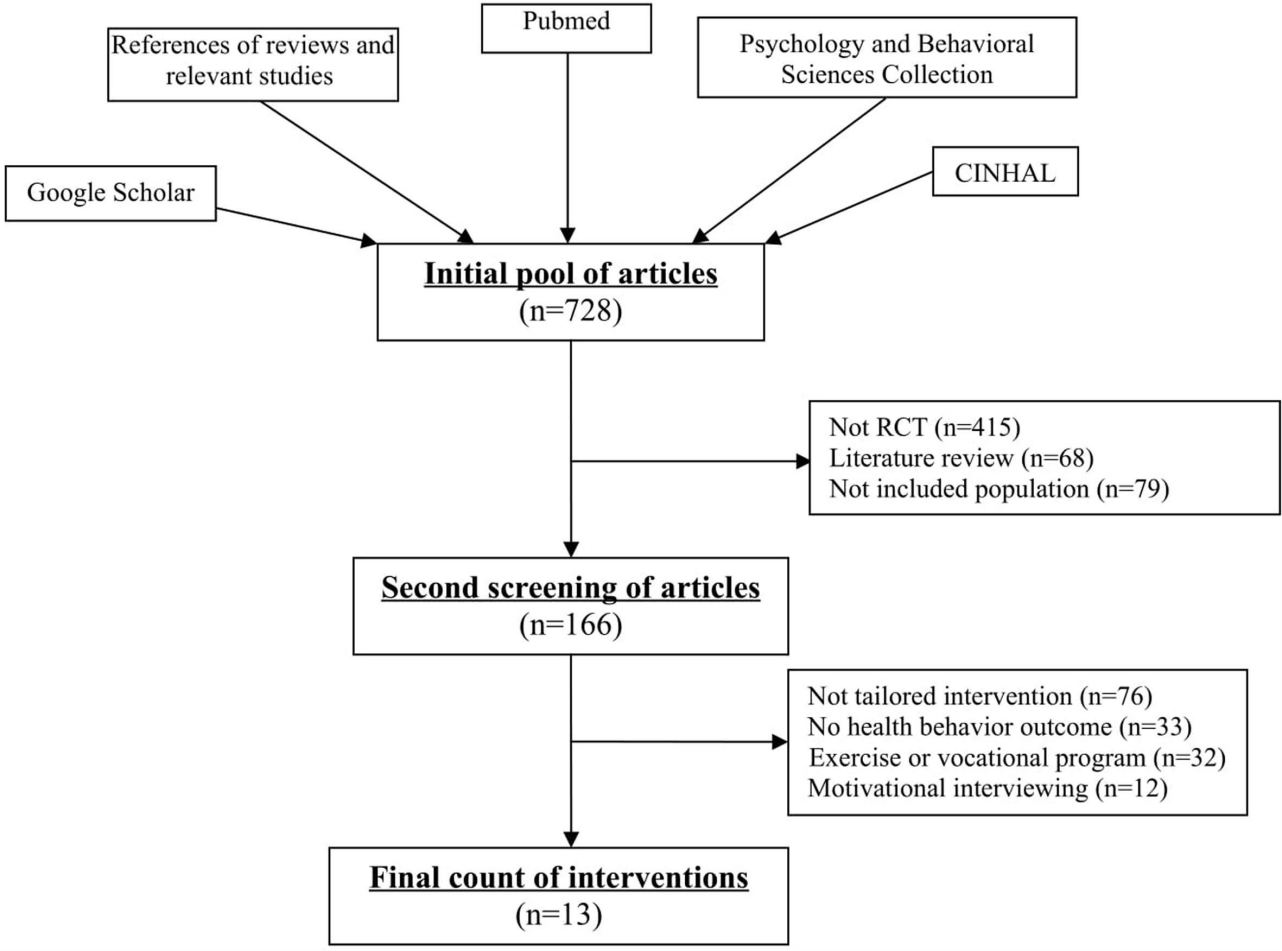
**Flow of articles through the study**.

We had an initial pool of 728 articles that described intervention strategies to promote healthy behaviors. Of these, we excluded 562 articles during the first phase of the review (see Figure 1). Articles were excluded for being literature reviews (*n* = 68), for not being RCTs (*n* = 415), or for not including adults with chronic neurological and musculoskeletal conditions living in the community (*n* = 79). In the second phase of review, we excluded additional articles that focused on the benefits of engaging in a particular type of exercise program or vocational rehabilitation program (*n* = 32); implemented an intervention that was not tailored to at least two personal characteristics (*n* = 76); failed to report the results of self-report questionnaires or sensors, such as an accelerometer, that measures the effects of the intervention on healthy behaviors (*n* = 33); or examined an intervention based on motivational interviewing (*n* = 12). A total number of 13 intervention studies met the inclusion–exclusion criteria.

#### Characteristics of the 13 Research Samples

Table 2 provides a summary of the characteristics for the 13 research samples. The included research samples had 4,184 community-dwelling adults with neurological and musculoskeletal conditions; 46% were females and 18.24% were non-white participants (reported from five studies). The weighted mean age was 52.5 years. Ten studies had research samples with a mean age of greater than 50. There were 1,034 adults with neurological conditions (stroke, *n* = 942; spinal cord injury, *n* = 62; and multiple sclerosis, *n* = 30), 1,193 adults with rheumatic diseases, and 689 adults with chronic pain.

**Table 2:** **Research design, outcomes, and description of intervention**.

References	*N*	Condition	Intervention topics	# of BCT	Delivery formats	Intervention length	Characteristics tailored on	Strategy to tailor content	Sig. outcomes
Basler et al. (33)	170	Low back pain	PA	4	One-to-one, in-person	5	Psychosocial const. Preferences Fun.	Judgment	
Bossen et al. (39)	199	Osteoarthritis	PA Pain	9	Web and phone	52	Preferences Impairments	Algorithm	Pain Fatigue Physical fun. Mental fun.
Eames et al. (34)	77	Stroke	PA Social fun. Mental fun. Physical fun. Nutrition Medication	3	One-to-one, in-person and phone	12	Preferences Impairments	Algorithm	Nutrition Medications
Evans-Hudnall et al. (35)	60	Stroke	PA Social fun. Mental fun. Physical fun. Nutrition Medication Smoking	16	One-to-one, in-person and phone	4	Demographics Barriers Psychosocial const. Risks	Judgment	Smoking Alcohol
Evers et al. (32)	64	Rheumatoid arthritis	Pain Fatigue Social fun. Mental fun. Physical fun.	14	One-to-one, in-person	52	Psychosocial const. Preferences Impairments	Judgment	Fatigue Mental fun. Social fun. Medication
Fries et al. (41)	1099	Osteoarthritis Rheumatoid arthritis	Pain Mental fun. Physical fun. Medication	4	Written material	52	Demographics Current behavior Barriers Psychosocial const. Fun.	Algorithm	Pain Physical fun. PA
Maasland et al. (36)	65	Stroke	PA Nutrition Medication Smoking Alcohol	3	Web	12	Demographics Risks Probability of outcome	Algorithm	
Murphy et al. (30)	42	Osteoarthritis	PA Fatigue Pain	6	One-to-one, in-person	10	Current behavior Impairments	Judgment	Fatigue Physical fun.
Plow et al. (31)	30	Multiple sclerosis	PA Fatigue Pain Social fun. Mental fun. Physical fun.	14	Written material	24	Current behavior Psychosocial const. Barriers	Algorithm	Physical fun. PA
Rimmer et al. (38)	92	Mobility impairments	PA	11	Group, in-person one-to-one, phone	24	Current behavior Barriers Psychosocial const. Preferences Fun. Impairments	Judgment	Weight Physical fun. PA
van der Ploeg et al. (37)	1202	Spinal cord Rheumatic disorders Stroke	PA	10	One-to-one, in-person and phone	14	Current behavior Barriers Psychosocial const. Preferences	Judgment	Physical fun. PA
Weymann et al. (42)	561	Low back pain	Pain Mental fun.	2	Web	12	Psychosocial const. Preferences	Algorithm	Mental fun.
Wolfe et al. (40)	523	Stroke	Smoking Alcohol Medication	2	One-to-one, in-person and written material	52	Current behavior Impairments Risks	Algorithm	

*BCT, behavior change techniques; Sig, significant; Fun., function; PA, physical activity; Const., Construct*.

There was little consistency on how health and function were characterized among the different research samples. However, some characteristics can be derived from the study criteria. For example, two studies excluded individuals for not being able to walk (30, 31), and eight studies excluded individuals because their disease status was assessed as being too severe (30–37). Three studies tried to avoid ceiling effects by excluding individuals whose disease status or symptom impact was assessed as being only minor (30, 31, 38).

#### Research Design and Outcomes

The majority of studies (*n* = 7) compared a tailored intervention to usual care or wait-list control condition (31, 32, 34, 35, 39–41). Four studies compared a tailored intervention to a non-tailored intervention (30, 33, 36, 42), and two studies had three comparator conditions to examine the effects of increasing the amount or intensity of intervention support (37, 38). Seven studies used single-blinded research designs; that is, they typically blinded the assessors (30, 31, 33, 34, 36, 37, 40). A total number of six studies conducted a power analysis to determine the appropriate sample size (30, 33, 35, 36, 40, 42), and eight studies reported conducting an intent-to-treat analysis (31–34, 37, 38, 40, 42). The attrition rate from pretest to the last measurement time point across all studies was 35.0%. No studies reported that participants experienced adverse events serious enough to cause them to withdraw from the study.

The timing of when outcome measures were administered in relation to the first pretest assessment varied greatly. The first posttest assessment ranged from 4 to 52 weeks (average 17 weeks) after the first pretest assessment. Five studies included two or more posttest assessments (31–33, 39, 41). The five studies included a second posttest assessment that ranged from 24 weeks to 52 weeks (average 41 weeks) after the first pretest assessment.

Outcome measures included patient-reported physical function (*n* = 8) (31, 32, 34, 35, 37–39, 41), and provider-reported/objective outcomes of physical function (*n* = 5) (30, 31, 33, 36, 39). Patient-reported outcome measures also included pain (*n* = 4) (30, 32, 39, 41), fatigue (*n* = 3) (30, 32, 39), mental/emotional health (*n* = 3) (32, 39, 42), and social function (*n* = 2) (32, 38). Health behavior outcomes included physical activity (*n* = 11) (30, 31, 33–39, 41, 42), smoking cessation (*n* = 4) (34–36, 40), alcohol use (*n* = 4) (34–36, 40), nutrition (*n* = 3) (34, 35, 42), and medication adherence (*n* = 3) (32, 34, 35).

Reported intervention effects on health and function included statistically significant improvements across time or between groups in patient-reported physical function (*n* = 5), fatigue (*n* = 3), patient-reported mental health (*n* = 3), provider-reported/objective outcomes of physical function (*n* = 2), pain (*n* = 2), and patient-reported social function (*n* = 1). Reported intervention effects on healthy behaviors included statistically significant improvements across time or between groups in physical activity (*n* = 4), medication adherence (*n* = 2), smoking cessation (*n* = 1), nutrition (*n* = 1), and alcohol use (*n* = 1). Eight studies included measures to examine why the intervention was or was not effective (31, 34, 35, 37–41), with seven of these studies exploring mechanisms of behavior change (e.g., exploring changes in self-efficacy) and one study exploring mechanisms for improving impairments in body functions and structures (39).

#### Description of Intervention

Details of the interventions can be found in Tables 2 and 3. The most common intervention topics included physical activity (*n* = 9), emotion management strategies (*n* = 6), and pain management strategies (*n* = 6). The most common delivery formats were face-to-face contacts (*n* = 9), either in a group (*n* = 1) or one-to-one instruction (*n* = 8). Six of the interventions that were delivered using face-to-face contacts also followed up with distance learning formats. Four interventions primarily used distance education approaches, that is, via Internet or phone.

Ten studies reported all three dosing parameters (i.e., duration, frequency, and amount). The duration of intervention ranged from 4 to 52 weeks (including follow-up visits). The most common intervention frequency was once per week or every other week (ranging from once per week to once every 3 months). The length of each intervention contact ranged from 5 to 60 min. The total number of contacts with the interventionist ranged from 0 to 30 contacts. Two interventions had an email/call-in service to answer participants’ questions when needed. A person who did not have a clinical license, typically was trained to deliver the interventions (e.g., a research assistant).

#### Behavior Change Techniques

Table 3 summarizes the frequency counts of the behavior change techniques used across all interventions. Five of the interventions were described as being based on a model or theory (31, 33, 34, 36, 37). The number of behavior change techniques used within a single intervention ranged from 2 to 16 techniques, with 7 interventions incorporating 5 or more behavior change techniques. The most common behavior change technique employed was presenting instructive information (*n* = 9), followed by feedback about performance (*n* = 8), self-monitoring of behavior (*n* = 7), action planning (*n* = 6), problem solving/barrier identification (*n* = 6), and stress management/emotional regulation (*n* = 6). Restructuring the environment (*n* = 1), training in communication skills (*n* = 1), and rewarding participants based on effort (*n* = 0) or success (*n* = 0) were infrequently or not applied behavior change techniques.

**Table 3:** **Overall characteristics of the 13 interventions**.

Variable	Count	Variable	Count
**Common intervention topics** Physical activity Emotional management Pain Physical function Medication adherence Smoking or alcohol use Social function Nutrition Fatigue Nutrition **Format of assessment to tailor content** Self-report questionnaire Oral history Objective measure Health record **Matching: characteristics tailored on** Psychosocial constructs Preferences Symptoms/impairments/conditions Current behavior Barriers Risk of adverse event Demographics Physical and mental function Probability of behavior change **Strategy used to tailor content** Clinical/expert judgment Computer algorithm	9 6 6 5 5 5 4 3 3 3 10 2 1 1 8 7 6 6 5 3 3 3 2 6 7	**Behavior change techniques** Instruction Feedback on performance Self-monitoring behavior Social support Problem solving Action planning Information on specific consequences Stress management Process goals Information performing behavior Follow-up prompts Relapse prevention Time management Review of process goals Demonstrate behavior Utilization of prompts Rehearsal and practice **Interventionist** Trained, non-clinical Occupational or physical therapists Physician/psychiatrist Personal Trainer	9 8 7 6 6 6 6 6 5 5 5 4 3 3 2 2 2 5 2 2 1

#### Tailoring Techniques

The types of variable used to match content to personal characteristics included psychosocial constructs (*n* = 8), preferences (*n* = 7), current behavior (*n* = 6), symptoms, impairments, or co-morbid conditions (*n* = 6), barriers (*n* = 5), demographic (*n* = 3), risk of an adverse event (*n* = 3), physical and/or mental function (*n* = 3), and probability of success (*n* = 1). Two interventions used objective biomarkers to tailor content. The format used to assess these characteristics typically was self-report questionnaires (*n* = 10). Oral history (*n* = 2), provider-reported assessments (*n* = 1), and health records (*n* = 1) were also used. Several interventions (*n* = 7) provided evaluative feedback and contextually framed content in terms of the participants’ age, culture background, physical environment, or types of impairments. However, none of the interventions were tailored by raising expectations or providing normative or progressive feedback. Seven interventions relied on algorithms to tailor content, and six studies used clinical judgment to tailor content.

## Discussion

Several factors are now facilitating opportunities to optimize and widely disseminate tailored self-management interventions: (1) advances in technology that are enabling the implementation of sophisticated computer algorithms that can guide the tailoring of ecological momentary interventions (EMIs), (2) big data analytics that can identify biomarkers on which to tailor interventions, and (3) sequential multiple assignment randomized trials (SMART) that can optimize decision rules for tailoring interventions (43). National research priorities also reflect opportunities to advance the science of tailoring. For example, National Institutes of Health’s newly created Precision Medicine Initiative seeks to identify biomarkers and lifestyle factors to account for individual variability in medical treatments (44). Methodological reports published by the Patient-Centered Outcomes Research Institute describe the importance of examining heterogeneity of treatment effect to better tailor interventions (45). Indeed, multiple stakeholders now have a vested interest in understanding how best to tailor interventions.

Thus, we have conducted the first literature review of tailored self-management interventions to promote healthy behaviors among adults with musculoskeletal and neurological conditions. Most of the studies were conducted in adults with chronic pain, stroke, and/or arthritis and few studies included more than one musculoskeletal or neurological condition. We found that none of the studies would have met the reporting standards for tailored interventions as recommended by Harrington and Noar (22). Our conclusions are consistent with other reviews of tailored behavior change interventions among healthy adults and adults with diabetes and cardiovascular disease (described in section below). To advance the science of tailoring self-management interventions, we recommend conducting comparative effectiveness research using big data analytics or employing a SMART design. There is also a need to further develop a taxonomy to standardize how tailoring strategies are described in self-management interventions.

### Comparison to Reviews of Tailored Interventions

Noar et al. (19) reported in a meta-analytic review that tailored print-based interventions in healthy adults were slightly better at changing healthy behaviors compared to control conditions. Heterogeneity in effect sizes could be explained based on differences in (a) dosing, (b) tailoring strategies, (c) comparison conditions, and (d) population segment (i.e., demographic characteristics). Unfortunately, our review of the literature does not provide any additional insights into why such factors may explain heterogeneity in effect sizes. We found that the descriptions of dosing and tailoring strategies were often vague, which makes it difficult to conduct meta-analyses or replicate the interventions. Although comparison conditions tested in RCTs provide some evidence that tailored interventions were effective, the comparisons themselves provide limited insight into why tailoring was effective or what are the optimal approaches to tailor content (46). In terms of population segments, Noar et al. stated that health literacy and socioeconomic status might be important factors to consider when designing tailored interventions. We found that the research samples included in our review were limited in cultural diversity, and few reported on socioeconomic status and race or ethnicity.

Radhakrishnan et al. (47) concluded in a literature review of 10 tailored self-management interventions among adults with type 2 diabetes, hypertension, and heart disease that tailored interventions may not be more effective than non-tailored interventions when cost and resource utilization are considered. Inclusion–exclusion criteria were similar to the criteria used in our study, including the definitions for tailoring. Generally consistent with our findings, Radhakrishnan et al. concluded that studies have “suffered from compromised methodological issues of inadequately powered sample size, non-blinding of data collection or intervention delivery and inadequate reporting of the randomization process.” We also note that few of the studies included in our review had long-term follow-up measures, including assessments of cost-effectiveness and quality of life outcomes related to social function. Because increased customization and segmentation may require higher implementation costs (15), it is important to document resource consumption in future research and demonstrate the long-term benefits in outcomes, such as healthcare utilization, sustained behavior change, and quality of life.

Richards et al. (17) found 63 articles that described RCTs of tailored interventions among healthy adults and adults with chronic conditions. The number of articles included in their review was higher than our present study due to differences in inclusion–exclusion criteria. For example, Richardson et al. considered tailoring to be an intervention that incorporated the unique characteristics of the person receiving care, including patient-centered or individualized interventions. We note that including interventions described as “patient-centered” or “individualized” would have substantially increased the number of articles in our review. However, we felt that “patient-centered” and “individualized” were not always consistent with Kreuter et al. (16) definition of a tailored intervention.

### Recommendations for Future Research

#### Mobile Health Technology

Mobile Health (mHealth) technology, such as smartphone and tablets, can deliver EMIs, which are highly tailored electronic messages delivered in peoples’ natural setting at a precise time to promote behavior change (48). Although studies in our review used phone calls and some used the Internet to interact with participants, none used EMIs. Many of the included studies may not have had access to affordable mHealth technology at the time of the study. mHeath technology is now more readily accessible and shows promise in delivering low cost EMIs to promote healthy behaviors in the general population (48). Thus, there is a need to examine whether mHeath technology delivering EMIs can improve the effectiveness of tailored self-management interventions in adults with neurological and musculoskeletal conditions. A potential fruitful area of research is using big data analytics to develop machine learning algorithms that deliver EMIs to promote self-management behaviors. The reader is referred to the following citations for further information on the topic (48–50).

#### Biomarkers

Most of the studies used self-report questionnaires about behavior to tailor intervention content. A few studies in our review used biomarkers of cardiovascular risk to tailor intervention content. As the field of big data analytics and Precision Medicine evolves, there will be greater opportunities to examine how biomarkers can be used to more precisely tailor self-management interventions beyond matching educational content to a particular cardiovascular risk factor. For example, can particular genotypes be used to tailor educational content to the learning style of the participant? There are now some promising lines of research in behavioral genetics in which education is tailored to optimize how students learn information (51, 52). Such research may be relevant for tailoring self-management interventions in adults with disabling conditions.

#### SMART Design

The growing acceptance of SMART designs also provides opportunities to advance the science of tailoring self-management interventions (43). A SMART design is a special type of factorial design that generates causal inference data to optimize decision rules on matching intervention content to individual characteristics. Furthermore, intervention strategies can be compared to each other in a SMART design to understand mechanisms of action or how tailoring works. A SMART design can compare delivery formats, dosing, and tailoring strategies to identify how to maximize quality of life using the available resources as efficiently as possible. For example, participants can first be randomized to receive tailored phone calls that have a high or low degree of customization and segmentation. Participants who do not respond to the phone calls or experience worsening of symptoms can be re-randomized to receive tailored in-person visits at low or high doses (e.g., comparing 3 visits to 6 visits). The reader is referred to the following citations for further information on the topic (43, 53–55).

#### Self-management Tailoring Taxonomy

We noticed in our scoping review of the literature that defining and conceptualizing tailoring was grounded in two distinct yet overlapping scholarly disciplines: health communication science and health care research. Both health communication scientists and health care researchers have described interventions existing on a continuum ranging from content that is completely standardized to content that is tailored to each participant (15, 17). However, health care research uses the term *tailoring* inconsistently and uses terms, such as *self-tailoring, patient-centered, personalized*, and *stepped care*, to describe interventions. Health communication scientists have precisely defined the term “tailoring” and have developed conceptual frameworks to describe tailored communications. However, terms used by health care researchers to describe tailoring have not been incorporated into conceptual frameworks of tailoring developed by health communication scientists. To advance the science of tailoring, there is a need to merge the conceptual frameworks of tailoring developed by health communication scientists with the conceptual frameworks of patient-centered care and self-management in health care research.

**Figure 2:**
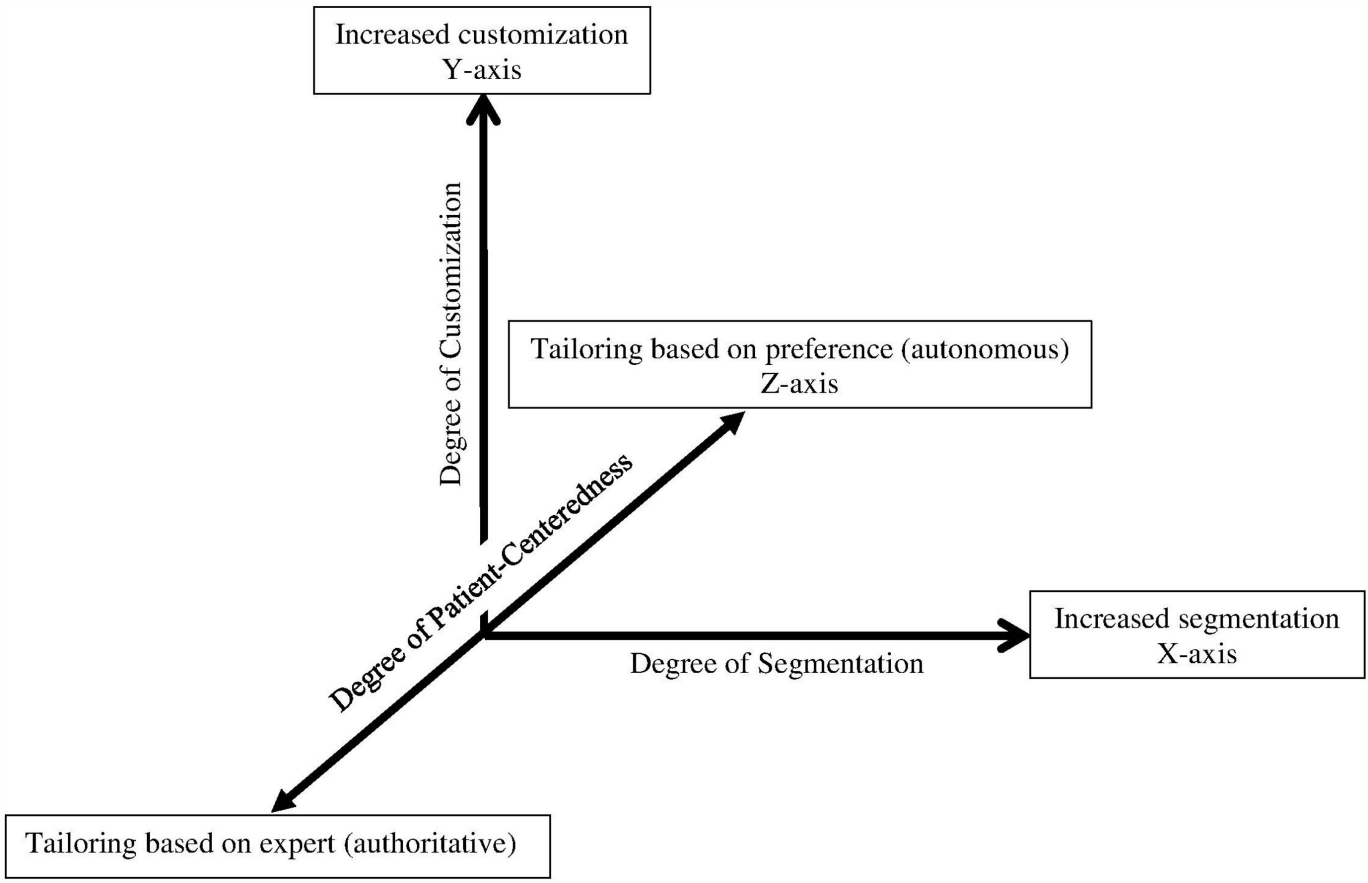
**Tailoring continuum to reflect the concept of patient-centered care: Adapted from Hawkins et al. (15)**.

For example, Hawking et al. (15) depicts customization on the *x*-axis and segmentation on the *y*-axis to show that as the number of homogenous groups increases, so does the extent of congruency between intervention content and individual characteristics. We propose that a *z*-axis needs to be considered to represent the extent to which the participant or the interventionist tailors the intervention content. At one end of the *z*-axis, the interventionist would decide entirely how to tailor content, that is, make recommendation without considering patient preferences (i.e., authoritative). On the other end of the *z*-axis, patients would make decisions on how to self-tailor content based on preferences, without limitation of options or content imposed by the health expert (i.e., autonomous). In the middle of the *z*-axis would lay patient-centered care, in which the interventionist and patient make mutual decisions, incorporating clinical expertise and patient preferences (see Figure 2).

In addition to merging conceptual frameworks from different disciplines, there is a need to further develop a taxonomy for tailoring strategies used in self-management interventions. We identified 4 types of assessment formats and 13 personal characteristics that were used to tailor self-management interventions. Assessment formats, personal characteristics based on which content is tailored, and the strategies used to tailor content, each, may have advantages and disadvantages and may differ with respect to resource consumption. To examine these advantages and disadvantages and the differences in resource consumption, a taxonomy is needed to clearly define and distinguish between tailoring strategies so that distinct comparisons can be made using a SMART design or big data analytics.

### Limitations

Several limitations to this review diminish our ability to draw generalizable conclusions about the most effective ways to tailor self-management interventions. Limitations include the unavailability of intervention manuals to code behavior change techniques, not calculating effect sizes, and not finding all the research studies that met the inclusion–exclusion criteria. We decided not to calculate effect sizes due to the small number of studies, the heterogeneity with which tailored interventions were implemented, and the heterogeneity in study outcomes. We were concerned that calculating effect sizes would lead to erroneous conclusions about which components of a tailored self-management intervention are effective. A SMART design would be a better way to examine the different components of tailoring and identify why tailoring is or is not effective.

We acknowledge that our review, although very comprehensive, may not have included all the interventions that met our criteria. However, the purpose of a scoping review is to summarize the breadth of existing literature rather than to calculate effect sizes to determine the magnitude of a particular intervention approach. Finally, we did not include articles that focused on motivational interviewing or evaluated the effects of exercise programs. Both types of interventions may be considered a type of tailored intervention. Thus, we refer the readers to review other articles for further information on motivational interviewing (56–59) and tailored exercise programs (60–62). We also refer the readers to a recently revised behavior change taxonomy that was not available at the start of this review (63). The taxonomy includes 93 behavior change techniques, but does not provide specific language to describe tailoring strategies.

## Conclusion

The 13 RCTs included in our review typically compared tailored interventions to non-tailored interventions or usual care. The tailored interventions were diverse in their delivery formats, dosing (i.e., duration, frequency, and amount), behavior change techniques, and tailoring strategies, but were focused mainly upon adults with chronic pain, stroke, and/or arthritis. Thus, there is a need to conduct further research of tailored self-management interventions in adults with other neurological and musculoskeletal conditions, such as multiple sclerosis, systemic lupus erythomatosis, Parkinson’s disease, fibromyalgia, and chronic fatigue syndrome. We recommend that a SMART design could be used to compare multiple aspects of tailoring to identify how best to promote self-management behaviors. We also recommend that future research should be conducted on mHealth technology to deliver highly tailored EMIs. It was clear from our review that a historical time context exists in that as technology advances so do tailoring strategies. As mHealth technology advances, it will be important to understand how to harness its potential to tailor self-management interventions. Finally, we recommend that future research is needed to further test and refine the preliminary tailoring taxonomy identified in this review.

## Author Contributions

MP formulated the primary research questions, interpreted the results, drafted the initial manuscript, and made critical revisions to the manuscript. SM, KG, and MG conducted the literature search, extracted the data from the articles, coded the articles, and made critical revisions to the manuscript. All authors read and approved the final manuscript.

## Conflict of Interest Statement

The authors declare that the research was conducted in the absence of any commercial or financial relationships that could be construed as a potential conflict of interest.
